# Beyond CREA: Evolutionary patterns of non‐allometric shape variation and divergence in a highly allometric clade of murine rodents

**DOI:** 10.1002/ece3.11588

**Published:** 2024-06-28

**Authors:** Ariel E. Marcy, D. Rex Mitchell, Thomas Guillerme, Matthew J. Phillips, Vera Weisbecker

**Affiliations:** ^1^ Commonwealth Scientific and Industrial Research Organisation (CSIRO), Science Connect Canberra Australian Capital Territory Australia; ^2^ College of Science and Engineering Flinders University Bedford Park South Australia Australia; ^3^ School of Biosciences University of Sheffield Sheffield UK; ^4^ School of Biology and Environmental Science Queensland University of Technology Brisbane Queensland Australia; ^5^ Centre of Excellence for Australian Biodiversity and Heritage Wollongong Australia

**Keywords:** allometry, CREA, geometric morphometrics, integration, modularity, Muridae, stabilising selection

## Abstract

The shared functions of the skull are thought to result in common evolutionary patterns in mammalian cranial shape. Craniofacial evolutionary allometry (CREA) is a particularly prominent pattern where larger species display proportionally elongate facial skeletons and smaller braincases. It was recently proposed that CREA arises from biomechanical effects of cranial scaling when diets are similar. Thus, deviations from CREA should occur with changes in cranial biomechanics, for example due to dietary change. Here, we test this using 3D geometric morphometric analysis in a dataset of Australian murine crania, which are highly allometric. We contrast allometric and non‐allometric variation in the cranium by comparing evolutionary mode, allometry, ordinations, as well as allometry, integration, and modularity in functional modules. We found evidence of stabilising selection in allometry‐containing and size‐free shape, and substantial non‐allometric variation aligned with dietary specialisation in parallel with CREA. Integration among cranial modules was higher, and modularity lower, with size included, but integration between rostrum and cranial vault, which are involved in the CREA pattern, dropped dramatically after size removal. Our results thus support the hypothesis that CREA is a composite arising from selection on cranial function, with substantial non‐allometric shape variation occurring alongside CREA where dietary specialisation impacts selection on gnawing function. This emphasises the need to research mammalian cranial evolution in the context of allometric and non‐allometric selection on biomechanical function.

## Graphical abstract

Rodent skulls display a highly conserved, strongly size‐dependent (allometric) range of shapes that is commonly found across mammals. We here use geometric morphometric analysis of Australian rodent cranial shape to show that this scaling is likely an emergent property of the specialised feeding biomechanics of most rodents, with other non‐size‐related processes also acting on adaptation of the skull.

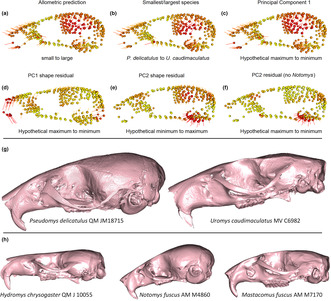

## 1 BACKGROUND

The skull is arguably the most functionally diverse interface between a mammal and its environment. It is employed in the acquisition and mastication of food, receives the majority of sensory input, and carries the large and heavy brain. The evolution of mammalian cranial diversity is therefore assumed to be heavily influenced by the various selection regimes acting on cranial function. Possibly for this reason, cranial morphology across mammals displays some common patterns of evolutionary variation. The most widely discussed of these is the tendency of larger mammals to have more elongate facial skeletons and smaller braincases relative to smaller species, particularly in closely related species (Cardini et al., 2015; Cardini & Polly, 2013; Mitchell, Potter, et al., 2024; Tamagnini et al., 2017). This pattern, termed craniofacial evolutionary allometry (CREA), exists in at least 11 vertebrate orders, especially those of mammals (Bright et al., 2016; Cardini, 2019), but the origins of this pattern have been unclear (e.g. Cardini, 2019).

Mitchell, Sherratt, & Weisbecker, (2024) suggested that CREA is likely a product of bite force allometry, phylogenetic niche conservatism, and potentially negative scaling of the braincase. Briefly, closely related species tend to obtain and process foods with similar mechanical properties, so that a large species needs to apply the same absolute bite forces to a food item as a small species. However, the mechanical demand on the cranium will be lower for larger species because of their larger size. Mitchell, Sherratt, and Weisbecker (2024) thus argued that larger species can sacrifice some capacity for bite force generation in response to other selective pressures, which could cause the more gracile facial skeletons that are part of the CREA pattern (Mitchell, Sherratt, & Weisbecker, 2024). Independently, the negative scaling of brain and orbit size will reduce the relative size of vault and orbital area, further increasing the appearance of facial elongation (Mitchell, Sherratt, & Weisbecker, 2024; e.g. Radinsky, 1985, among many others).

Many studies of mammalian cranial shape evolution are consistent with the concept of CREA as a result of biomechanics (e.g. Cardini et al., 2015; Figueirido et al., 2013, 2014; Tamagnini et al., 2017, 2023), and the effect was recently demonstrated in a genus of marsupial rock‐wallabies (Mitchell, Potter, et al., 2024). However, the view in Mitchell, Sherratt, and Weisbecker (2024) suggests that CREA reflects one part of biomechanical evolution as far as it pertains to allometry, but that changes in dietary regime will result in shape changes not captured by CREA. This is indeed the case for a range of mammals covered in Mitchell, Sherratt, and Weisbecker (2024) and particularly well documented in felids (Tamagnini et al., 2017, 2023). The biomechanical insights that can be gained from the CREA pattern are therefore not complete if non‐allometric processes of selection also contribute to cranial shape variation.

Rodents are an excellent test case for exploring the interface between CREA and non‐allometric cranial shape variation because this clade is among the most striking cases of allometry coinciding with a CREA pattern. A previous study (Marcy et al., 2020) showed that a sample of mostly Australian rodents, diverging as early as 10 million years ago, has a highly conserved slope of allometry explaining over a third of their overall shape variation. The shape variation explained by allometry is also aligned with CREA (Marcy et al., 2020; Mitchell, Sherratt, & Weisbecker, 2024). This is probably due to the specialisation of the rodent jaw to gnawing, which is highly conserved (Cox et al., 2012) but allows the clade substantial dietary breadth (Druzinsky, 2015; Marcy et al., 2020; Zelditch & Swiderski, 2023). Within the clade, similar cranial specialisations can arise from different developmental growth patterns (Segura et al., 2023; Wilson, 2013). This supports the suggestion that CREA arises from stabilising selection on cranial function, rather than alternative hypotheses that it represents intrinsic developmental constraints (e.g. Cardini & Polly, 2013). A conserved, allometric ‘one‐to‐many’ mapping of cranial function might also explain the clade's unique overall shape but slow morphological evolution through time (Goswami et al., 2022), and finds that rapid and extensive changes in shape, such as island gigantism, are possible within species along the allometric line (Schlis‐Elias & Malaney, 2022).

In addition to the strong CREA pattern, and consistent with Mitchell, Potter, et al.'s (2024); Mitchell, Sherratt, and Weisbecker's (2024) predictions, Marcy et al. (2020) noted that species whose shape departed most from the common evolutionary allometric pattern tended to be ecological specialists with distinct diets and locomotor modes. Examples include the broad‐toothed rat *Mastacomys fuscus* with exceptionally broad molars (Ford, 2006), whose diet nearly entirely consists of grass (Calaby & Wimbush, 1964; Green et al., 2014) which is highly abrasive to dentition (e.g. Winkler et al., 2019). Another deviation from the common allometric line is seen in the two carnivorous sister species, the Rakali (*Hydromys chrysogaster*) and water mouse (*Xeromys myoides*), and a group of ecological specialists with distinct non‐diet‐related locomotor modes, the hopping mice (*Notomys*) and the rabbit‐rat (*Conilurus penicillatus*). The hopping mice are of interest because of their conspicuous ‘facial tilt’ of the anterior cranium, an adaptation resulting in an expansion of their field of view while hopping or bounding (Kraatz & Sherratt, 2016).

The clear departures of some ecological specialists from the common allometric line make Marcy et al.'s (2020) dataset an excellent test case to assess whether the CREA pattern is just the allometric pattern among a diverse range of biomechanical selection pressures, which need to be investigated jointly by studies into mammalian cranial evolution. In this study, we test several predictions arising from this hypothesis by contrasting between allometric and non‐allometric shape variation (via analysis of residuals from regressions of shape against size) on Marcy et al.*'s* (2020) sample of Australian murines.

A main prediction of a biomechanically driven CREA pattern is that it should be bounded by functional allometric optimisation (Mitchell, Sherratt, & Weisbecker, 2024). This should also limit the amount of shape divergence of cranial shape through time (e.g. Beaulieu et al., 2012), so that even remotely related species should display similar shapes. This should result in an Ornstein‐Uhlenbeck pattern of shape divergence through time, where limited diversification occurs around an allometrically determined optimal shape (Harmon et al., 2010). Furthermore, if non‐allometric variation is also under stabilising selection due to other biomechanical selection acting in parallel to allometry‐generating selection, similar bounded divergence over time should arise in the residual cranial variation.

In addition, the biomechanical processes leading to CREA should be reflected in high allometry and high levels of co‐variation in cranial areas that are expected to vary most under CREA as described above – the rostrum, cranial vault, and potentially orbital region (Cardini et al., 2015; Cardini & Polly, 2013; Mitchell, Sherratt, & Weisbecker, 2024). By contrast, selection on functions that result in shape variation independent of CREA is expected to be more apparent in some parts of the skull but not others depending on cranial function (e.g. the maxillary region of carnivorous species; the back of the skull in hopping or bounding species). We test these hypotheses by comparing ordination and landmark variation plots of cranial shape data versus allometric residuals. Furthermore, we use assessments of cranial integration (covariation between modules; Klingenberg, 2009) and modularity (the degree of independence of shape variation within a module relative to the others; Klingenberg, 2009) to understand how the co‐variation between different parts of the cranium changes in datasets with and without size. Strong allometry in the rodent sample means that the skull co‐evolves with size as one integrated structure, so that the integration between modules (i.e. their co‐variance; Bookstein, 2015; Klingenberg, 2009) is expected to be higher and modularity (i.e. independence of modules from each other) potentially lower in datasets with shape information contained. Integration should also be strongest between those modules identified as affected by CREA. In combination, these results will help map a way forward of how evolutionary change in mammalian cranial morphology can be interpreted in joint allometric and non‐allometric frameworks.

## 2 METHODS

We used Marcy et al.'s (2020) previously published dataset of 37 Australian rodent species (averaged from shapes of a total of 317 individuals) that were landmarked with 60 fixed landmarks, 141 curve semi‐landmarks, and 124 patch semi‐landmarks. These were subjected to a generalised Procrustes analysis (GPA) with subsequent removal of the asymmetric component (details in Marcy et al., 2020 and implemented in the github repository associated with this study). Ecological information on diet and locomotion for each species was taken from Breed and Ford (2007).

All analyses were performed in R (v.4/3/3; R Core Team, 2024), using mostly the packages *geomorph* v. 4.0.7 (Adams et al., 2022; Baken et al., 2021), mvMORPH v. 1.1.9 (Clavel et al., 2015), *landvR* v. 0.5.2 (Guillerme & Weisbecker, 2019), *phytools* v. 2.1‐1 and *vegan* v. 2.6‐4 (Oksanen et al., 2022). To compare allometric and modularity patterns, we separated landmarks according to a five‐module framework that followed the six modules proposed across therian mammal crania (Goswami, 2006). This included the anatomical regions of the rostrum, molar area, orbital area, vault, basicranial area, but excluded the zygomatic arch module, which was missing due to scanner limitations (Marcy et al., 2018). For the purposes of assessing allometric variation independently, the raw data of these modules were also subjected to the same processing steps (GPA with subsequent asymmetry removal) as the full raw data.

### 2.1 Evolutionary modes

To assess whether the crania in our sample follow an Ornstein‐Uhlenbeck (OU) pattern of evolution, as predicted by our hypothesis of stabilising selection, we used mvMORPH to fit models of Brownian motion (BM), Ornstein‐Uhlenbeck (OU), and also Early Burst (EB). The EB test computes a scenario of rapid initial radiation with subsequent decrease in diversification (Clavel et al., 2015). We also fitted GLS (generalised least square) models of allometry under the three evolutionary scenarios to find out which evolutionary mode fits the evolution of allometry best (BM, OU or EB), and additionally the most likely mode of evolution of the residuals of that model. To identify the best modes, we compared the generalised information criterion for each of the fits by calculating their relative probabilities (*W* scores; Burnham & Anderson, 2002). Lastly, we also investigated the most likely evolutionary mode that explained the observed distribution of the log‐transformed centroid size through by calculating W scores of the Akaike information criterion (AIC) outputs (using the Phytools package).

Note that the relatively small sample size (*n* = 37) reduces the confidence of estimations of evolutionary modes (Cooper et al., 2016), and hence we preferred to use residuals of the more conventional BM‐based models of allometry for our downstream analyses. To ensure that this was acceptable, we used two‐block partial least squares (2B‐PLS) analysis to confirm that shape residuals from our BM and OU models were similar (i.e. have a high r‐PLS correlation score). This test is also important to understand if substantial differences in results could be expected in our downstream analyses of integration and modularity, which are only available in *geomorph* in the context of Brownian motion models.

### 2.2 Visualising shape evolution through phylo‐morphological distance plots

As noted above, the sample size available to us (*n* = 37 species) makes estimations of evolutionary modes potentially unreliable (Cooper et al., 2016). We therefore also visualised change of shape relative to time by plotting the Procrustes distances between species against evolutionary time. For this, we retrieved a matrix of pairwise phylogenetic distances using the *picante* function *cophenic* (Kembel et al., 2010) on our ultrametric time‐calibrated phylogeny (Marcy et al., 2020; Smissen & Rowe, 2018). Because the tree did not contain any fossils, values were divided by two to express them in millions of years since last common ancestor. The pairwise Procrustes distances – that is, morphological distances – were derived from the GPA of shapes. We then plotted every pairwise combination of the phylogenetic and morphological distances between two species in our dataset for both the full shape and shape residual datasets. We expected this to provide a broad estimate of morphological divergences with and without allometry, but there are two caveats to this method: (1) pseudoreplication due to the high volume of pairwise comparisons within the sample and (2) non‐uniform sampling of time due to the phylogeny's structure, with most coverage occurring between 0.3 and 4.2 Ma. We therefore interpret the results with these caveats in mind.

### 2.3 Comparing the distribution of species in morphospace through PCA scores

To visually assess distribution of species in the allometric and non‐allometric morphospaces, we performed principal component analyses (PCA) on three different shape datasets of mean species shapes, and visualised the two first dimensions of each morphospace (PC1 and PC2). The first morphospace, termed here ‘full shape dataset’ is based on a conventional generalised Procrustes analysis (GPA), and includes the allometric component of shape. The second, the ‘residual dataset’, includes the components of shape that remain once allometric shape is removed and it provides a ‘size‐less’ or ‘allometry‐free’ comparison of the species shapes. The shape residuals were obtained from a phylogenetically informed linear generalised least squares model using random permutations implemented by the *RRPP* package (Collyer & Adams, 2018, 2019). When residuals were added to the consensus shape derived from the GPA, the shape variation could be compared visually to the full shape dataset. For the third morphospace, we repeated the PCA for the shape residual dataset after removing the four hopping mice (genus *Notomys*). We did this because we expected their bipedal posture to exaggerate some features of shape variation in the PCA and the resulting morphospace plots. Lastly, the phylogenetic signal contained in centroid size has the potential to remove relevant size information. We therefore also computed a supplementary PCA and heat plots of shape change based on residuals from a simple linear model.

### 2.4 Assessment of allometric versus allometry‐free shape variation via heat maps

To visualise and assess allometric shape variation in the full shape dataset, we created heatmaps showing the magnitude of landmark displacements using *landvR* functions (Guillerme & Weisbecker, 2019; Weisbecker et al., 2019). We compared three different visualisations of allometry. First, using fitted allometric shapes estimated by Procrustes linear models (also using random permutations as per RRPP) across the entire sample. However, variation characterised through ordination or allometric analysis provides summaries of parts of the variation, which do not always reflect actual specimens (Weisbecker et al., 2019). We therefore also visualised the mean configurations of the smallest native species (the delicate mouse, *Pseudomys delicatulus*) and the largest (the giant white‐tailed rat, *Uromys caudimaculatus*), as determined by mean centroid size. Third, to assess the similarity in shape variation along PC1 to the two previous visualisations, we visualised the hypothetical shapes for PC1 minimum and maximum. Lastly, we present meshes from our samples to allow assessments of how well the quantitative patterns reflect biological realities.

To compare the allometric shape change to the ‘isometry‐free' (i.e. scaled during superimposition) or ‘allometry‐free’ shape variation, we produced heatmaps from the shape residual dataset visualising the minimum and maximum hypothetical shapes for three different PC axes. First, we produced heatmaps for PC1 and PC2 to compare the allometry‐free changes to the allometric cranial changes seen in the full shape dataset. We also visualised heatmaps for the shape residual PC2 without the four species of *Notomys* to assess the impact of their bipedal posture on the ordinated shape variation. Again, we presented meshes of species of particular interest to allow comparison with the landmark variation presentations.

### 2.5 Allometry, modularity and integration

Because CREA is expected to affect the rostral, cranial vault, and possibly orbit areas, we tested if size explained more variation in these three compared to the molar and basicranial modules. These analyses can be done after separate GPA/asymmetry removals for each module; however, this risks not capturing allometric size change between modules, which is an important part of our question. We therefore report the results of allometry analyses of shape versus log‐transformed centroid size for each module based on joint GPA and separate GPAs, each with an asymmetry removal step included.

The impact of allometry on shape variation was further assessed by comparing integration (degree of co‐variation) and modularity (degree of module independence) across the cranium and between modules before and after size removal. Integration was measured using partial least squares (PLS) correlation coefficients between multiple modules, taking into account phylogeny (Adams & Felice, 2014); values towards 1 indicate a higher PLS coefficient. To quantify modularity, we used *geomorph* functionalities (Adams, 2016; Adams & Collyer, 2019) to calculate the covariance ratio (CR) coefficient, with the numerator as covariation between modules and the denominator as covariation within modules. Therefore, highly modular structures, with higher covariation within than between modules, will have small covariance ratio values within the unit interval. By contrast, structures with low modularity will have CR values close to 1.0 because the two covariation values are very similar (Adams, 2016). In both modularity and integration analyses, the functions include a phylogenetic context by generating a matrix of partial least squares under a Brownian motion model of evolution (Adams & Felice, 2014) that was informed by our time‐calibrated ultrametric molecular phylogeny (Marcy et al., 2020). The resulting evolutionary covariance matrix controls for similarities between closely related species, which is needed to study macro‐evolutionary patterns of modularity (Adams & Felice, 2014; Klingenberg & Marugán‐Lobón, 2013). Significance was determined by randomly resampling the modules 1000 times and comparing the random distribution of coefficients to the observed value. We also compared the R‐PLS and CR values among modules in the full versus the residual dataset.

To assess if removal of the integrating influence of allometry results in reduction in cranial integration, we also conducted comparisons of integration strengths between modules in the full versus the residual dataset as implemented in *geomorph*; these derive statistical significances from comparisons between effect sizes of pairs of PLS analyses (Adams & Collyer, 2016). As with the allometry analyses, we expected the areas most affected by CREA (rostrum, braincase, and possibly orbits) to be more integrated with each other than the remainder of the modules. We tested this expectation by comparing the level and relative strength of integration for all pairs of modules as outlined above, and again also asked if integration is reduced significantly between pairs of modules in the full versus the residual dataset (Adams & Collyer, 2019).

The CR‐coefficient‐based modularity analyses in geomorph are designed to detect significant modular structure under a specific hypothesis of modularity (Adams & Collyer, 2019); unlike integration analyses, modularity comparisons are therefore not designed to compare differences in the level of modularity between different datasets such as ours, which have the same hypothesised modular structure. To still obtain an assessment of whether individual modules are more independent of each other (i.e. modular) after size correction, we performed pairwise Mantel tests on the distance matrices of PC scores within each module (Legendre & Legendre, 2012). The resulting *r* statistic indicates the degree of correlation between each module pair, with values closer to one corresponding to higher integration (Hetherington et al., 2015). If a module consistently has *r* statistics closer to zero, this indicates higher modularity, that is, greater independence in shape variation relative to the other cranial modules. Note that this analysis has the caveat of being without phylogenetic adjustment. The Bonferroni correction was used to adjust for multiple comparisons (Bonferroni, 1936).

## 3 RESULTS

### 3.1 Evolutionary mode

Generalised least squares (GLS) models of shape variation alone, shape allometry, and residual shape were most likely under the assumption of Ornstein‐Uhlenbeck evolution; in all cases, the OU model had a*W*‐score of 1 compared to 0 for the Brownian motion (BM) and Early Burst (EB) models. By contrast, a Brownian motion model was more likely for the evolution of size, although both OU and EB models also have a moderate likelihood (Table 1).

**TABLE 1:** Generalised/Akaike information criterion scores (GIC/AIC) and *W*‐scores of relative probabilities of modes of evolution for models of shape, log‐ transformed centroid size, and shape evolution with log (centroid size) as predictor variable.

DONE	Brownian motion	Ornstein‐Uhlenbeck	Early burst
Shape GIC	−446,170	**−447,701**	−446,167
Shape W	0	**1**	0
Shape~log (Csize) GIC	−433,056	**−434,260**	−433,054
Shape~log (Csize) W	0	**1**	0
Shape residuals GIC	−475,814	**−476,364**	−475,812
Shape residual W	0	**1**	0
Log (Csize) AIC	**−4.802**	−2.875	−2.802
Log (Csize) W	**0.572**	0.218	0.21

*Note*: The most likely modes are bolded.

As OU‐structured evolutionary modes are not implemented in our analyses of modularity and integration, which are based on BM, we further asked how well the residuals of the OU versus BM models of shape and allometry evolution corresponded by comparing residual coordinates of shape from both models using two‐block partial least squares. In both cases, the r‐PLS value was 1, suggesting that no substantial difference to BM‐based analyses is to be expected. We thus also proceeded with allometric residuals of BM‐based allometric models.

### 3.2 Visualising shape evolution through phylo‐morphological distance plots

**FIGURE 1:**
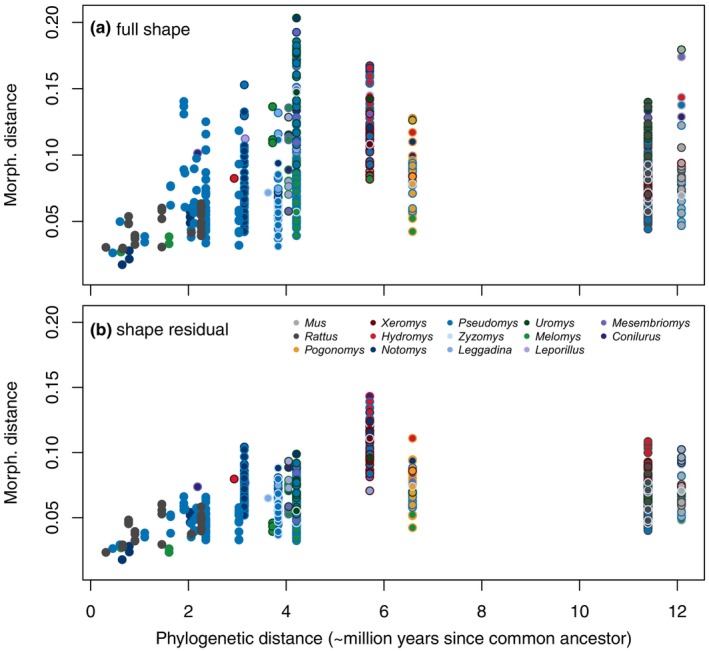
Phylo‐morphological pairwise distances plots. Each point is a pairwise comparison with border and centre colours corresponding to the two species' genera. The x‐axis is shared but the y‐axes of morphological distances are not equivalent as they rely on different shape datasets: (a) full shape and (b) shape residual.

Our phylo‐morphological distance plots (Figure 1) examined whether phylogenetic time‐since‐divergence and morphological distances (i.e. Procrustes distances between the mean shapes of a species pair) increase with increasing phylogenetic distance because, as integration patterns change over time, shape covariation patterns can diverge (Voje et al., 2014). As expected, all of the points closest to the origin (i.e. low phylogenetic *and* low morphological distances) are within‐genus pairs. In the full shape dataset, maxima in morphological distances tend to increase with phylogenetic distance until reaching an asymptote around 4.2 Ma since the last common ancestor. However, the highest divergence values involve distances of all species with the two large‐bodied frugivores: *Uromys caudimaculatus* and the black‐footed tree rat *Mesembriomys gouldii* (Figure 1a). If these are ignored, then the morphological distances appear to plateau earlier, around 2 Ma. Furthermore, shape distances between *Rattus* and other Australian murines, which have divergence dates of around 10 million years, fall well within the range of morphological distances within murines. However, as noted in methods, these results are subject to pseudoreplication because they include all pairwise combinations, such that each of the 37 species accounts for 36 data points. This can be seen in the vertical clustering, which represent pairwise comparisons between one species and other species with the same divergence time.

The ‘allometry‐free’ shape residual pairwise comparisons were similar to the full shape dataset, with overall lower morphological distances as expected from removing allometric shape variation. The removal of allometric differences between species also has a marked effect on the spread of morphological distances at each divergence. Most conspicuously, removing allometry substantially reduces morphological distances between the large‐bodied frugivores relative to other ecological specialists, so that the greatest distances between species now correspond to the divergence between the two semiaquatic, carnivorous species at 5.7 Ma (Figure 1b). If the semiaquatic species are ignored, the dataset's remaining maximum distances appear around 3.1 Ma, or pairwise comparisons between hopping *Notomys* species and close relatives in *Pseudomys*. Both plots show the greatest morphological divergences occurring within the old endemic species, not between more distantly related species pairs involving *Rattus* or *Mus*.

### 3.3 Comparing the distribution of species in morphospace through PCA scores

**FIGURE 2:**
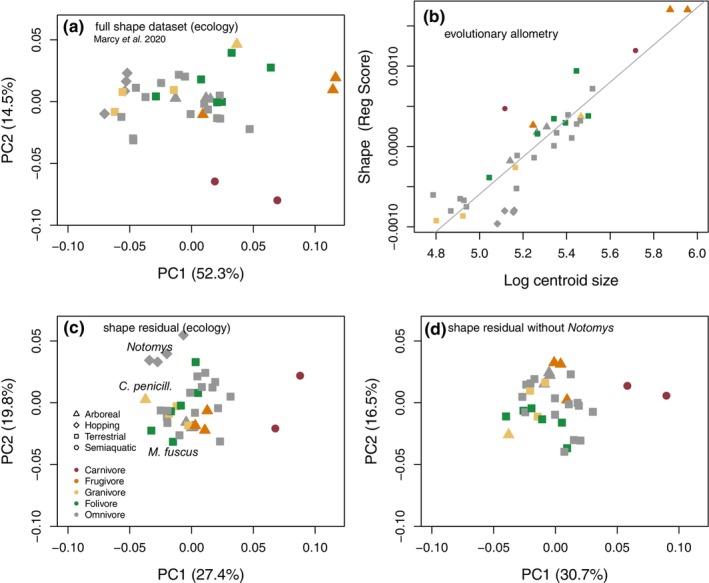
Shape variation related to size and after size removal. (a) Plot of PC1 and PC2 for the full shape dataset and (b) plot of log centroid size versus the projected regression score with a grey regression line indicating the common evolutionary trajectory as per Marcy et al. (2020); (c) ‘allometry‐free’ shape residual dataset with *Notomys* genus, *Conilurus penicillatus*, and *Mastacomys fuscus* highlighted; (d) shape residual dataset without *Notomys*, which mainly just switches the sign of the PC scores.

Comparing the variation and species distribution from the first two principal components (PC) of the full and residual datasets shows that the removal of allometric shape variation substantially reduces the amount of variation in the dataset that is aligned with PC1. PC2 axes captured similar percentages of shape variation (note, however, that the overall amount of variation is smaller for the residual dataset so that less variation is captured in the residual PC2). As expected, the full shape PC1 orders species by size (with a correlation of PC1 to size of 0.92). The species distribution along the full shape PC2 resembles the pattern along the residual shape PC1 (Figure 2a vs c) in that both axes show the carnivorous *Xeromys myoides* and *Hydromys chrysogaster* at one extreme and a quadrupedal bounding species (the brush‐tailed rabbit rat, *Conilurus penicillatus*) at the other. Removal of size thus mostly removes the shape information of PC1 from the residual dataset, with PC2 of the full dataset correlating at 0.97 with PC1 of the residual dataset. Similarly, a mantel test of the distance matrices between species derived from the full‐dataset PCA without PC1 versus the distance matrices from PCA of the residual dataset showed a very high correlation (0.94). This shows that size removal does not impact much on the distribution of shape variation beyond PC1. The distinctive shape of the cranium of *Notomys*, arising from its bipedal posture, was not a main driver of residual shape variation: when *Notomys* was removed, the relative positioning of species and the shape variation associated with the first two PCs remain similar (Figure 2c vs d).

In the shape residual plot of PC1 and PC2, the majority of species cluster in the centre. This includes the two large‐bodied frugivores, whose shape lies on the common line of allometry (Figure 2b). The allometry‐free PC plots separate out other ecological specialists instead, such as the two semiaquatic carnivores along PC1 and the four hopping *Notomys* species along PC2 (Figure 2c). The broad‐toothed rat (*Mastacomys fuscus*), which is a specialised consumer of grasses, is separated by low residual PC2 values.

### 3.4 Assessment of allometric versus allometry‐free shape variation via heat maps

**FIGURE 3:**
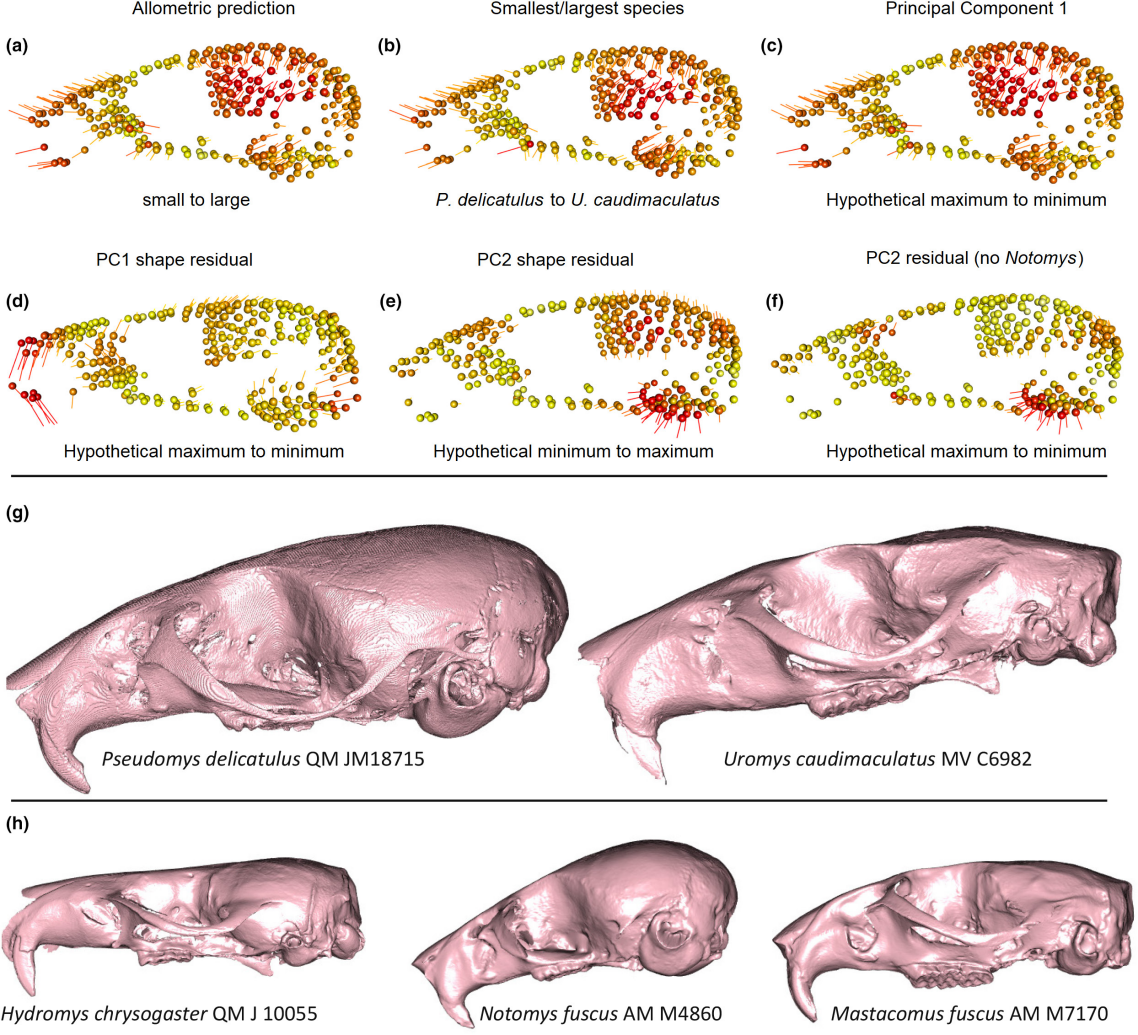
(a) shape differences between the shape fitted for mean centroid size of the smallest to the largest species in the sample; (b) shape differences between the mean shapes of these two species; (c) differences between the hypothetical shapes captured between PC1 extremes; (d) differences between the hypothetical shapes captured between PC1 extremes based on allometry‐free data; (e) differences between the hypothetical shapes captured between PC2 extremes on allometry‐free data; (f) differences between hypothetical shapes between PC1 extremes after removing *Notomys*. Spheres show the mean position of landmarks for the column's dataset, vectors show landmark displacement. Colours and lengths are calculated from relative proportions of the minimum/maximum vector lengths for each comparison and are not equivalent across individual images. (g) comparison between the smallest versus largest species in the sample (reflecting the variation seen in 3a–c); (h) examples of species on extremes of residual PC1 (*H. chrysogaster*), and residual PC1/2 (*N. fuscus*, *M. fuscus*), crania aligned so the foramen magnum is approximately vertical. Not to scale. *P. delicatulus* specimen is from Marcy et al. (2018).

The expected CREA pattern of relatively longer rostra and smaller brain cases with size is apparent in the visualisation of shape variation that is associated with allometry (Figure 3a–c). This is also clearly visible in comparisons of mean shapes between smallest and largest species in the dataset and predicted shapes for high and low PC1 scores for the full dataset. Comparing meshes of representatives from the smallest and largest species provides visual confirmation that the allometric and ordinated variation reflects major differences between the crania (Figure 3g) and is not an artefact of Procrustes superimposition. Comparison with the shape variation captured by residuals from a non‐phylogenetically corrected linear model of shape relative to log(centroid size) shows very few differences both in the amount of variation and shape change explained by PC1 and PC2 (Figure S1).

As expected, removing the shape variation that covaries with size (Figure 3d–f) also removed the CREA‐aligned patterns. Species closer to the PC1 maximum, such as the carnivorous *H. chrysogaster* (Figure 3h) then show a straighter anterior rostra/incisor regions and dorsoposterior displacement of the foramen magnum (Figure 3d,h). However, the residual PC2 heatmaps highlight shape patterns resembling individual aspects of CREA‐like variation in the cranial vault, but without associated variation in the rostrum. For example, the *Notomys* species at PC1 minimum show dorsally expanded braincases and ventrally expanded auditory regions, but not shortened rostra as expected under CREA (Figure 3e and mesh in 3h). This coincides with other differences, like variation in the dorsal maxillary region (3e, compare *Notomys* and *Mastcomys* in Figure 3h). Removing the four bipedal hopping species of *Notomys* reduced this pattern somewhat to highlight just the expansion of the bulla, but as with the PC1/2 plots of Figure 1, the result showed similar regions of variation (Figure 3f). This indicates that the bipedal hoppers, despite their distinctive morphology, do not dominate the variation in both PCAs.

### 3.5 Allometry, modularity and integration

The amount of variation explained by size varied widely among modules (Table 2), and had larger effect sizes and *R* ^2^ values in the joint GPA compared to separate GPAs. Surprisingly, the rostral module was revealed as having the least of its variation explained by size, despite its extensive variation predicted by allometric fit heatmaps (Figure 3); in fact, the association between rostral shape and rostral centroid size was just below the significance threshold.

**TABLE 2:** Phylogenetically adjusted generalised least squares analysis (PGLS) of module shape versus log‐transformed centroid sizes for joint (top) and separate (bottom) GPAs.

	SS	MS	*R* ^2^	*F*	*Z*	*p*
Joint GPA						
Basicranium	0.002	0.133	.13	5.38	3.31	**.000**
Molar	0	0.09	.09	3.47	2.7	**.002**
Orbital	0.001	0.206	.21	9.07	3.59	**.000**
Rostrum	0.003	0.074	.07	2.79	2.29	**.007**
Vault	0.01	0.263	.26	12.49	3.79	**.000**
Separate GPA						
Basicranium	0.01	0.085	.09	3.26	2.69	**.002**
Molar	0.025	0.139	.14	5.63	3.74	**.000**
Orbital	0.027	0.196	.2	8.51	3.37	**.000**
Rostrum	0.005	0.038	.04	1.37	1.44	.077
Vault	0.021	0.184	.18	7.9	4.14	**.000**

Abbreviations: *F*, *F* values; MS, mean squares; *p*, *p*‐value (probability of significant association at *p* < .05 based on 10,000 permutations); *R* ^2^, *R*‐squared value; SS, sum of squares; *Z*, *Z* scores (effect sizes from *F* values).

Thresholds for significance were set at p=0.05

**FIGURE 4:**
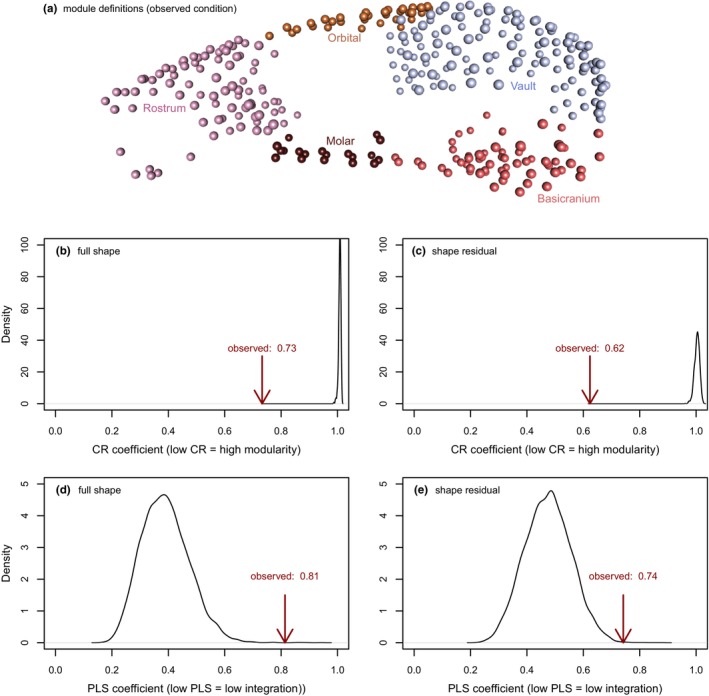
Modularity and integration results averaged across the whole skull (a) our five‐module framework (Goswami, 2006. Results from the full shape (b,d) and shape residual (c,e) datasets. Black curves are the density distribution of coefficients from 1000 randomly drawn modules and the arrows point to the observed coefficients, which were all significant.

As expected, the full dataset had higher levels of integration (high PLS correlation coefficient) and lower modularity (CR coefficient closer to 1) than the shape residual dataset (Figure 4) because it contains the co‐variation of shape with size. However, despite the clear drop in value, the strength difference between the full and residual dataset was just outside the significance cut‐off (*p* = .066; effect size of 1.83). As we also predicted, size‐independent patterns of shape evolution exist in parallel with allometric variation of shape, with greater independence of the cranial modules suggested by the lower r‐PLS and CR coefficients of the shape residual dataset.

Assessment of pairwise integration between modules revealed that integration between the rostrum and the cranial vault was the highest among all module pairs (Table 3, lower triangle). Unlike the allometry analyses, this is consistent with the allometric pattern on rostral elongation with relative reduction of the vault (refer to Figure 3). Furthermore, the orbital region has high integration values relative to both rostrum and vault, consistent with our prediction that orbital size might also play a role in the evolution of allometric variation. Strong co‐variation between rostrum and basicranium is notable and was not part of our predictions.

**TABLE 3:** Phylogenetically informed integration, expressed as r‐PLS values, between the full shape (lower triangle) and residual shape (upper triangle) of modules.

	Basicranium	Molar	Orbital	Rostrum	Vault
Basicranium	64	0.59↓	0.8↑	0.71↓	0.88↑
Molar	0.69	19	(0.52)↓	0.82↑	0.69↓
Orbital	0.77	0.70	32	0.76↓	0.85↓
Rostrum	0.89	0.77	0.86	86	0.79↓
Vault	0.85	0.77	0.88	0.94	124

*Note*: All r‐PLS values were significant at *p* < .05 except for the integration between residuals of orbital and molar modules. For *p*‐value tables, see Table S1. Arrows in the upper triangle indicate whether the r‐PLS values of residual integration analyses are higher (↑) or lower (↓) than the full shape r‐PLS values. Numbers in the diagonal are landmark numbers for each partition.

Removal of size resulted in several changes in r‐pls values (Table 3, upper triangle), but not all of these related to reduced integration and only some were detected as significant differences in integration strength (Table 4). All instances of significant integration strength differences relate to the vault with all other modules. The greatest difference in effect size reflects a dramatic drop in integration between the vault and the rostrum, followed by a more moderate drop in r‐PLS value between vault and orbits and vault and molars. Intriguingly, an *increase* in integration between vault and basicranium is also detected as a significant change in integration strength, while a very large drop in R‐pls value between rostrum and basicranium is just outside the significance cut‐off (*p =* .06).

**TABLE 4:** Integration strength comparisons between pairs of modules, including effect sizes (*Z*) and significance of strength differences (*p*).

	*Z* full	*Z* residual	*Z* difference	*p*
Basicranium × molar	3.34	2.25	0.86	.388
Basicranium × orbital	4.16	4.1	0.63	.526
Basicranium × rostrum	4.29	2.98	1.88	.06
**Basicranium** × **vault**	3.93	4.89	2.11	**.035**
Molar × orbital	3.09	1.44	1.23	.217
Molar × rostrum	3.81	4.24	0.45	.654
**Molar** × **vault**	3.69	2.74	2.1	**.036**
Orbital × rostrum	4.45	3.43	1.37	.17
**Orbital** × **vault**	4.1	4.22	2.41	**.016**
**Rostrum** × **vault**	4.94	3.19	2.48	**.013**

Thresholds for significance were set at *p*= .05

In contrast to the mixed changes in integration patterns, modularity among all cranial partitions increased after the removal of size (Table 5), with no apparent difference as to how much integration levels change or whether there is a significant change in integration strength after size removal. This is also reflected in our Mantel tests comparison of distance matrices between species according to their PCA scores (Table 6); while remaining nearly all significant (meaning that the distribution of species in PC morphospaces remains more similar than expected at random), all partition comparisons had lower Mantel r statistics after the removal of size, with exception of the rostrum/molar comparison which remained nearly unchanged in both the CR‐based modularity assessment (Table 5) and the Mantel tests (Table 6).

**TABLE 5:** Phylogenetically informed modularity, expressed as CR coefficients, between the full shape (lower triangle) and residual shape (upper triangle) of modules.

	Basicranium	Molar	Orbital	Rostrum	Vault
Basicranium	64	0.52	0.5	0.63	0.73
Molar	0.56	19	0.46	0.66	0.62
Orbital	0.66	0.6	32	0.66	0.74
Rostrum	0.82	0.68	0.81	86	0.72
Vault	0.82	0.69	0.84	0.86	124

*Note*: Numbers on the diagonal are landmark numbers for each partition.

**TABLE 6:** Modularity tests using pairwise Mantel comparisons of PCA‐based distance matrices of all modules, and Mantel *r* statistic.

	Basicranium	Molar	Orbital	Rostrum	Vault
Basicranium	64	0.355 (.03)	0.290 (.07)	0.508 (.01)	0.674 (.01)
Molar	0.514 (.01)	19	0.351 (.03)	0.658 (.01)	0.424 (.02)
Orbital	0.601 (.01)	0.55 (.01)	32	0.620 (.01)	0.606 (.01)
Rostrum	0.719 (.01)	0.625 (.01)	0.807 (.01)	86	0.694 (.01)
Vault	0.753 (.01)	0.564 (.01)	0.731 (.01)	0.762 (.01)	124

*Note*: An *r* statistic of 1 indicates a strong correlation and 0 indicates no correlation. The upper triangle reports statistics for pairwise comparisons between cranial modules of the full dataset, and the lower triangle reports *r* statistics for the residual dataset. The values in brackets are *p* values, adjusted by Bonferroni (1936) corrections for multiple comparisons.

## 4 DISCUSSION

In this study, we sought to understand the degree to which non‐allometric shape variation occurs in the highly allometric clade of Australian murine rodents and their more phylogenetically remote invasive relatives. The comparison of datasets with and without size‐related shape variation confirms that size variation represents just one of several sources of variation, with substantial non‐allometric signatures of shape divergence, ordinated variation, integration, and modularity.

Allometry in mammalian crania, and the associated shape variation as predicted by CREA, has often been attributed to the integration of size with masticatory biomechanics (Marroig & Cheverud, 2005; Mitchell et al., 2018; Mitchell, Potter, et al., 2024; Singleton, 2005; Weisbecker et al., 2019). This is probably particularly true for rodents, where high levels of allometry likely reflect constraints imposed by their highly derived gnawing function (Cox et al., 2012; Druzinsky, 2015; Ginot et al., 2018; Lessa & Patton, 1989; Marcy et al., 2016, 2020). The greater likelihood of an Ornstein‐Uhlenbeck (OU) pattern of limited diversification around a local optimum (Harmon et al., 2010) reinforces this impression, particularly because size itself most likely evolves according to BM. However, the OU mode of evolution is also most likely in our ‘allometry‐free’ dataset of residuals, which anticipates the finds of our downstream analyses that allometric variation is only one manifestation of the overarching impact of biomechanical adaptation on the rodent cranium.

Plotting morphological divergences against time also shows a tendency for cranial shape divergence to plateau around 2 million years ago among species that retain their ancestral ecological niche. By contrast, here are several ‘spikes’ of morphological divergence where a substantial change in either size or cranial function evolved. In the full shape dataset, this corresponds to the evolution of unique – but allometrically expected – shapes of the large‐bodied frugivores (sensu Schluter; Marcy et al., 2020). Changes of lesser magnitude are then revealed by the allometry‐free data, where divergences are greatest when the dietary shift to carnivory selected for a fundamentally different masticatory action (Freeman & Lemen, 2008; Satoh & Iwaku, 2006), and where the hopping *Notomys* display a change in the genus‐level allometric multidimensional intercept (discussed further below).

The OU‐patterned evolution of shape in allometric and allometry‐free contexts is also reflected in the fact that the cranium and all but one pair of modules remain significantly and sometimes slightly more integrated after removal of allometry. This is also reflected in the lack of significance when comparing integration strength between the full and residual dataset, which was just beyond the significance cut‐off. The high integration between the rostrum and the cranial vault in the full dataset is predicted by CREA, and is notable because it supports the hypothesis of Mitchell, Potter, et al. (2024); Mitchell, Sherratt, and Weisbecker (2024) that CREA is a mosaic arising from different mechanisms; in rodents, this would be bite force allometry affecting the rostral area combined with hypo‐allometry of the brain affecting the cranial vault. Consistent with CREA patterns being driven mostly by allometry, the integration between the vault and the rostrum also drops most dramatically after the removal of size. However, the mostly significant integration levels after size removal confirm our expectation that allometric patterns are just one manifestation of cranial adaptation and do not represent a singular constraining process, and that other sources of covariation clearly shape cranial variation in Australian rodents. This would also explain why the modularity after size removal does not mirror changes in integration, as differences in functional adaptation (i.e. size‐related versus size‐unrelated, in our case) can lead to a shift between the two (e.g. Felice & Goswami, 2018; Ferreira‐Cardoso et al., 2022).

As already discussed in Marcy et al. (2020), the allometric prediction and variation among PC1 extremes support the existence of a CREA pattern of rostral elongation and relative reduction of braincase size. However, the gracilisation that is part of the expected CREA pattern is not as apparent in our sample as it is in other mammals (Mitchell, Potter, et al., 2024; Mitchell, Sherratt, & Weisbecker, 2024) because the rostral elongation coincides with a dorsal expansion of the rostrum. Visual assessment of the cranial meshes in Figure 3 suggests that this might be a unique feature of rodent cranial allometry related to the ever‐growing incisors, which extend much further into the dorsal rostrum compared to other mammals and are known to dominate the rostral shape of rodents (Marcy et al., 2016; McIntosh & Cox, 2016).

While our ordinations, visualisations of shape change, and integration results are all consistent with the existence of a CREA pattern, an unexpectedly low (even non‐significant, in separate GPAs) amount of variation in the rostrum is attributable to size. Interpreting this result represents a challenge and we can only offer some suggestions here. One possibility is that the visualisations of Procrustes‐superimposed landmark variation are impacted by the ‘Pinocchio‐Effect’, where variation in the tip of a triangular shape – such as most vertebrate rostra – is exaggerated (summarised in Klingenberg, 2021). However, this should also affect the statistical analyses of the rostral module in the joint GPA; moreover, comparisons of cranial meshes in Figure 3 demonstrate that differences in rostral shape between small and large species are real and substantial.

If the low allometry of the rostrum reflects biological reality, there are several potential explanations for this effect. It is probably relevant that the rostrum's extensive non‐allometric variation (e.g. on residual PC1) reflects its greater diversity in function, making an allometric effect relatively small compared to partitions like the cranial vault, whose variation is predominantly explained by size. If the substantial allometric component of vault variation is strongly integrated with the less extensive allometric component of rostrum variation, this would explain the high integration between vault and rostrum, and emphasis on rostral shape variation in the heat plot visualisations. It is also possible that the function of the rostral region is associated with isometric size variation, which Procrustes analysis like ours does not capture. Such an effect is predicted when the musculature changes or a species' bite force matches the increase in bite force associated with being larger (Mitchell, Sherratt, & Weisbecker, 2024). In murids, this seems to be the case, as a study of 14 species showed isometric increase in‐vivo bite forces (Ginot et al., 2018). It is therefore possible that increases in size result in limited trade‐offs between size and rostral gracility in murine rodents, potentially because of the scaling of incisors noted above.

The Australian murines in our sample display relatively few dietary specialisations, which prevented statistical analyses of their influence on cranial variation. However, the distribution of these species' cranial shape in the full and residual shape morphospaces is consistent with known hypotheses of mammalian cranial biomechanics. The carnivorous rakali (*Hydromys chrysogaster*) and water mouse (*Xeromys myoides*), whose crania are least adapted to the extensive gnawing action typical of other rodents (Cox et al., 2012), show high residual PC1 scores reflecting their straight, elongate incisor regions. This results in a much wider gape, consistent with the benefits of larger gapes in carnivorous rodents (Hennekam et al., 2020; Satoh & Iwaku, 2006; Williams et al., 2009) and specifically the rakali (Fabre et al., 2017). The departure from the common allometric line thus reflects the carnivorous species' functional shift from omnivory to carnivory, which is thought to occur at the expense of bite force at the molars (Fabre et al., 2017); a similar departure from CREA due to a larger gape has also been observed in sabre‐toothed cats (Tamagnini et al., 2023). By contrast, the specialised grass‐feeding broad‐toothed rat *Mastacomys* and granivore rabbit‐rat *Conilurus penicillatus* score low on both the residual PC1 and 2, reflecting curved anterior rostra and relatively smaller cranial vaults. This is consistent with findings of the wider skulls and dorsally shifted temporalis muscles that increase the muscle mass for masticating fibrous foods, which has evolved in specialist folivores across several rodent families (Samuels, 2009) and leads to more robust cranial dimensions in this species (Breed & Ford, 2007) and other folivores (Barbero et al., 2023). Similarly, cranial morphology is expected to be strongly influenced by the most challenging foods encountered by a species (Figueirido et al., 2014; Mitchell, 2019; Strait et al., 2009; Van Valkenburgh, 1989), so that frequent consumption of hard seeds and insects by the desert‐living hopping generalists *Notomys* and *Conilurus* (Murray et al., 1999) might explain their more robust crania relative to braincase than expected for their size. This adds the dietary specialists among our sample to the other cases of shape divergence from the allometric line that can be explained by cranial biomechanics (e.g. Mitchell, Sherratt, & Weisbecker, 2024; Tamagnini et al., 2017, 2023).

Another, potentially non‐biomechanical effect emerging from the PCA of shape residuals is a tendency of the dorsal cranial vault to expand dorsally together with a ventral expansion of the basicranium on residual PC2, resulting in overall braincase expansion. This may be related to changes in the proportions between cranium and brain size, either through increase or decrease in encephalisation (Smaers et al., 2021) or a different distribution of brain tissue within the braincase (Weisbecker et al., 2021). The genera for which this effect is most obvious – *Mastacomys* (with a relatively small braincase) and *Notomys* (with a relatively large braincase) indeed have large residuals, despite slopes of static (within‐species) allometry that are not significantly different from the common slope (Marcy et al., 2020). The changes in braincase dimension thus appear to reflect a ‘grade shift’ of an otherwise identical allometric pattern. This further supports the expectation that bite force allometry should be expressed in similar patterns in crania with divergent shapes (e.g. represented by different intercepts of an allometric regression; Mitchell, Sherratt, & Weisbecker, 2024).

Despite evidence that the allometric pattern in our sample is determined by stabilising selection on a particular biting pattern, the allometry‐free morphospaces show that this appears not to constrain the evolution of adaptations such as postural variation coinciding with ecological specialisations. For example, the rabbit rat (*Conilurus penicillatus*) has the highest facial tilt of the sample, consistent with its quadrupedally bounding locomotion (Kraatz & Sherratt, 2016; Watts & Kemper, 1989) However, despite its unusual shape, the rabbit rat still falls along the common allometric line, thus suggesting that stabilising selection on mastication permits the evolution of specialist postures. A similar pattern is seen in the bipedally hopping genus *Notomys*, which is second in facial tilt to *Conilurus*. *Notomys* species do not lie on the common allometric line, but this separation is because of their basicranium and vault shape, not their facial tilt. The inclusion of a facial tilt in *Conilurus* and *Notomys* within the common allometric pattern therefore confirms the hypothesis that CREA – patterns are related to specific parts of the cranium, without representing a constraint on the entire skull.

## 5 CONCLUSIONS

Characterising the allometric and allometry‐free shape variation in the cranium of Australian murine rodents has provided a useful context to recent suggestions that allometric shape variation is a biomechanics‐driven process among many (Mitchell, Sherratt, & Weisbecker, 2024). Most importantly, CREA emerges as an important explanation for cranial integration, but assessments of size‐free variation reveal it as just one out of several patterns that are well explained by established biomechanical hypotheses. Another important insight is that some patterns of postural adaptation, in our case relating to facial tilt, appear to be integrated with a common allometric line, producing a shared evolutionary shape pattern for the majority of the diverse sample. However, deviations from the allometric line and OU‐patterned residual variation also support suggestions that size is only a constraint where stabilising selection for a particular cranial function is apparent. This explains why, among the many homogenous cranial shapes of rodents, striking deviations occur with biomechanical changes, for example, in mastication musculature (for example, as seen in hystricomorphs) or extreme dietary shifts occur (such as in worm‐specialists like Paucidentomys; Esselstyn et al., 2012). Our results thus highlight how CREA is well explained as an emergent property of several sub‐patterns, which can differ among clades depending on a range of scaling. This makes observations of CREA by themselves of limited use for understanding the evolution of cranial shape in mammals. Adding the nuance required for assessing craniofacial allometry and other biomechanical processes in mammals will therefore pose a challenge for future studies of cranial shape variation.

## AUTHOR CONTRIBUTIONS

**Ariel E. Marcy:** Conceptualization (equal); data curation (lead); formal analysis (equal); investigation (equal); methodology (equal); project administration (equal); visualization (lead); writing – original draft (lead); writing – review and editing (supporting). **David Rex Mitchell:** Investigation (equal); validation (equal); writing – review and editing (equal). **Thomas Guillerme:** Formal analysis (equal); methodology (equal); validation (equal); writing – review and editing (equal). **Matthew J. Phillips:** Conceptualization (supporting); funding acquisition (supporting); supervision (equal); writing – original draft (supporting); writing – review and editing (equal). **Vera Weisbecker:** Conceptualization (lead); formal analysis (lead); funding acquisition (lead); investigation (equal); methodology (lead); supervision (equal); visualization (supporting); writing – original draft (supporting); writing – review and editing (lead).

## CONFLICT OF INTEREST STATEMENT

The authors declare that they have no competing interests.

### OPEN RESEARCH BADGES

This article has earned Open Data and Open Materials badges. Data and materials are available at 10.5281/zenodo.11022305 and https://www.morphosource.org/projects/00000C561.

## Supporting information


Data S1.

## Data Availability

The dataset of 3D specimen scans on which the landmarks are based are available on MorphoSource (https://www.morphosource.org/projects/00000C561). The dataset of landmark coordinates and the fully reproducible code for the analyses in the current study are available on DOI 10.5281/zenodo.11022305. The meshes plotted in Figure 3 are available on 10.25451/flinders.25662387.

## References

[ece311588-bib-0001] Adams, D. C. (2016). Evaluating modularity in morphometric data: Challenges with the RV coefficient and a new test measure. Methods in Ecology and Evolution, 7(5), 565–572. 10.1111/2041-210X.12511

[ece311588-bib-0002] Adams, D. C. , & Collyer, M. L. (2016). On the comparison of the strength of morphological integration across morphometric datasets. Evolution, 70(11), 2623–2631. 27592864 10.1111/evo.13045

[ece311588-bib-0003] Adams, D. C. , & Collyer, M. L. (2019). Comparing the strength of modular signal, and evaluating alternative modular hypotheses, using covariance ratio effect sizes with morphometric data. Evolution, 73(12), 2352–2367. 10.1111/evo.13867 31657008

[ece311588-bib-0004] Adams, D. C. , Collyer, M. L. , Kaliontzopoulou, A. , & Baken, E. K. (2022). Geomorph: Software for geometric morphometric analyses. R package version 4.0.4. https://cran.r‐project.org/package=geomorph

[ece311588-bib-0005] Adams, D. C. , & Felice, R. N. (2014). Assessing trait covariation and morphological integration on phylogenies using evolutionary covariance matrices. PLoS One, 9(4), e94335. 10.1371/journal.pone.0094335 24728003 PMC3984176

[ece311588-bib-0006] Baken, E. K. , Collyer, M. L. , Kaliontzopoulou, A. , & Adams, D. C. (2021). Geomorph v4.0 and gmShiny: Enhanced analytics and a new graphical interface for a comprehensive morphometric experience. Methods in Ecology and Evolution, 12, 2355–2363. 10.1111/2041-210X.13723

[ece311588-bib-0007] Barbero, S. , Teta, P. , & Cassini, G. H. (2023). An ecomorphological approach to the relationship between craniomandibular morphology and diet in sigmodontine rodents from central‐eastern Argentina. Zoology, 156, 126066. 10.1016/j.zool.2022.126066 36563591

[ece311588-bib-0008] Beaulieu, J. M. , Jhwueng, D.‐C. , Boettiger, C. , & O'Meara, B. C. (2012). Modeling stabilizing selection: Expanding the Ornstein–Uhlenbeck model of adaptive evolution. Evolution, 66(8), 2369–2383. 10.1111/j.1558-5646.2012.01619.x 22834738

[ece311588-bib-0009] Bonferroni, C. (1936). Teoria statistica delle classi e calcolo delle probabilita. Pubblicazioni del R Istituto Superiore di Scienze Economiche e Commericiali di Firenze, 8, 3–62.

[ece311588-bib-0010] Bookstein, F. L. (2015). Integration, disintegration, and self‐similarity: Characterizing the scales of shape variation in landmark data. Evolutionary Biology, 42(4), 395–426. 10.1007/s11692-015-9317-8 26586921 PMC4642606

[ece311588-bib-0011] Breed, B. , & Ford, F. (2007). Native Mice and Rats.

[ece311588-bib-0012] Bright, J. A. , Marugán‐Lobón, J. , Cobb, S. N. , & Rayfield, E. J. (2016). The shapes of bird beaks are highly controlled by nondietary factors. Proceedings of the National Academy of Sciences, 113(19), 5352–5357. 10.1073/pnas.1602683113 PMC4868483 27125856

[ece311588-bib-0013] Burnham, K. P. , & Anderson, D. R. (2002). Model selection and multi‐model inference. Springer.

[ece311588-bib-0014] Calaby, J. , & Wimbush, D. (1964). Observations on the broad‐toothed rat, *Mastacomys fuscus* Thomas. CSIRO Wildlife Research, 9(2), 123–133.

[ece311588-bib-0015] Cardini, A. (2019). Craniofacial allometry is a rule in evolutionary radiations of placentals. Evolutionary Biology, 46(3), 239–248. 10.1007/s11692-019-09477-7

[ece311588-bib-0016] Cardini, A. , Polly, D. , Dawson, R. , & Milne, N. (2015). Why the long face? Kangaroos and wallabies follow the same ‘rule’ of cranial evolutionary allometry (CREA) as placentals. Evolutionary Biology, 42(2), 169–176. 10.1007/s11692-015-9308-9

[ece311588-bib-0017] Cardini, A. , & Polly, P. D. (2013). Larger mammals have longer faces because of size‐related constraints on skull form. Nature Communications, 4(1), 2458. 10.1038/ncomms3458 24045342

[ece311588-bib-0018] Clavel, J. , Escarguel, G. , & Merceron, G. (2015). mvMORPH: An R package for fitting multivariate evolutionary models to morphometric data. Methods in Ecology and Evolution, 6(11), 1311–1319.

[ece311588-bib-0019] Collyer, M. L. , & Adams, D. C. (2018). RRPP: An R package for fitting linear models to high‐dimensional data using residual randomization. Methods in Ecology and Evolution, 9, 1772–1779. 10.1111/2041-210X.13029

[ece311588-bib-0020] Collyer, M. L. , & Adams, D. C. (2019). RRPP: Linear Model Evaluation with Randomized Residuals in a Permutation Procedure. https://CRAN.R‐project.org/package=RRPP

[ece311588-bib-0021] Cooper, N. , Thomas, G. H. , Venditti, C. , Meade, A. , & Freckleton, R. P. (2016). A cautionary note on the use of Ornstein Uhlenbeck models in macroevolutionary studies. Biological Journal of the Linnean Society, 118(1), 64–77. 27478249 10.1111/bij.12701 PMC4949538

[ece311588-bib-0022] Cox, P. G. , Rayfield, E. J. , Fagan, M. J. , Herrel, A. , Pataky, T. C. , & Jeffery, N. (2012). Functional evolution of the feeding system in rodents. PLoS One, 7(4), e36299. 10.1371/journal.pone.0036299 22558427 PMC3338682

[ece311588-bib-0023] Druzinsky, R. E. (2015). The oral apparatus of rodents: Variations on the theme of a gnawing machine. In L. Hautier & P. G. Cox (Eds.), Evolution of the rodents: Advances in phylogeny, functional morphology and development (Vol. 5, pp. 323–349). Cambridge University Press.

[ece311588-bib-0024] Esselstyn, J. A. , Achmadi, A. S. , & Rowe, K. C. (2012). Evolutionary novelty in a rat with no molars. Biology Letters, 8(6), 990–993. 10.1098/rsbl.2012.0574 22915626 PMC3497122

[ece311588-bib-0025] Fabre, P. H. , Herrel, A. , Fitriana, Y. , Meslin, L. , & Hautier, L. (2017). Masticatory muscle architecture in a water‐rat from Australasia (Murinae, Hydromys) and its implication for the evolution of carnivory in rodents. Journal of Anatomy, 231(3), 380–397. 28585258 10.1111/joa.12639 PMC5554825

[ece311588-bib-0026] Felice, R. N. , & Goswami, A. (2018). Developmental origins of mosaic evolution in the avian cranium. Proceedings of the National Academy of Sciences, 115(3), 555–560. 10.1073/pnas.1716437115 PMC5776993 29279399

[ece311588-bib-0027] Ferreira‐Cardoso, S. , Claude, J. , Goswami, A. , Delsuc, F. , & Hautier, L. (2022). Flexible conservatism in the skull modularity of convergently evolved myrmecophagous placental mammals. BMC Ecology and Evolution, 22(1), 87. 10.1186/s12862-022-02030-9 35773630 PMC9248141

[ece311588-bib-0028] Figueirido, B. , Tseng, Z. J. , & Martín‐Serra, A. (2013). Skull shape evolution in durophagous carnivorans. Evolution, 67(7), 1975–1993. 23815654 10.1111/evo.12059

[ece311588-bib-0029] Figueirido, B. , Tseng, Z. J. , Serrano‐Alarcón, F. J. , Martín‐Serra, A. , & Pastor, J. F. (2014). Three‐dimensional computer simulations of feeding behaviour in red and giant pandas relate skull biomechanics with dietary niche partitioning. Biology Letters, 10(4), 20140196. 10.1098/rsbl.2014.0196 24718096 PMC4013707

[ece311588-bib-0030] Ford, F. (2006). A splitting headache: Relationships and generic boundaries among Australian murids. Biological Journal of the Linnean Society, 89(1), 117–138.

[ece311588-bib-0031] Freeman, P. W. , & Lemen, C. A. (2008). A simple morphological predictor of bite force in rodents. Journal of Zoology, 275(4), 418–422. 10.1111/j.1469-7998.2008.00459.x

[ece311588-bib-0032] Ginot, S. , Herrel, A. , Claude, J. , & Hautier, L. (2018). Skull size and biomechanics are good estimators of in vivo bite force in murid rodents. The Anatomical Record, 301(2), 256–266. 10.1002/ar.23711 29330946

[ece311588-bib-0078] Goswami, A. (2006). Cranial modularity shifts during mammalian evolution. The American Naturalist, 168(2), 270–280. 10.1086/505758 16874636

[ece311588-bib-0033] Goswami, A. , Noirault, E. , Coombs, E. J. , Clavel, J. , Fabre, A.‐C. , Halliday, T. J. D. , Churchill, M. , Curtis, A. , Watanabe, A. , Simmons, N. B. , Beatty, B. L. , Geisler, J. H. , Fox, D. L. , & Felice, R. N. (2022). Attenuated evolution of mammals through the Cenozoic. Science, 378(6618), 377–383. 10.1126/science.abm7525 36302012

[ece311588-bib-0034] Green, K. , Davis, N. , & Robinson, W. (2014). Diet of the broad‐toothed rat *Mastacomys fuscus* (Rodentia:Muridae) in the alpine zone of the Snowy Mountains, Australia. Australian Zoologist, 37(2), 225–233. 10.7882/az.2014.023

[ece311588-bib-0035] Guillerme, T. , & Weisbecker, V. (2019). LandvR: Tools for measuring landmark position variation. *Zenodo*. 10.5281/zenodo.2620785

[ece311588-bib-0036] Harmon, L. J. , Losos, J. B. , Davies, T. J. , Gillespie, R. G. , Gittleman, J. L. , Jennings, W. B. , Kozak, K. H. , McPeek, M. A. , Moreno‐Roark, F. , Near, T. J. , Purvis, A. , Ricklefs, R. E. , Schluter, D. , Schulte, J. A., II , Seehausen, O. , Sidlauskas, B. L. , Torres‐Carvajal, O. , Weir, J. T. , & Mooers, A. Ø. (2010). Early bursts of body size and shape evolution are rare in comparative data. Evolution, 64(8), 2385–2396. 10.1111/j.1558-5646.2010.01025.x 20455932

[ece311588-bib-0037] Hennekam, J. J. , Benson, R. B. J. , Herridge, V. L. , Jeffery, N. , Torres‐Roig, E. , Alcover, J. A. , & Cox, P. G. (2020). Morphological divergence in giant fossil dormice. Proceedings of the Royal Society B: Biological Sciences, 287(1938), 20202085. 10.1098/rspb.2020.2085 PMC7735280 33143584

[ece311588-bib-0038] Hetherington, A. J. , Sherratt, E. , Ruta, M. , Wilkinson, M. , Deline, B. , & Donoghue, P. C. J. (2015). Do cladistic and morphometric data capture common patterns of morphological disparity? Palaeontology, 58(3), 393–399. 10.1111/pala.12159

[ece311588-bib-0039] Kembel, S. W. , Cowan, P. D. , Helmus, M. R. , Cornwell, W. K. , Morlon, H. , Ackerly, D. D. , Blomberg, S. P. , & Webb, C. O. (2010). *Picante*: R tools for integrating phylogenies and ecology. Bioinformatics, 26(11), 1463–1464. 10.1093/bioinformatics/btq166 20395285

[ece311588-bib-0040] Klingenberg, C. P. (2009). Morphometric integration and modularity in configurations of landmarks: Tools for evaluating a priori hypotheses. Evolution and Development, 11(4), 405–421. 10.1111/j.1525-142X.2009.00347.x 19601974 PMC2776930

[ece311588-bib-0041] Klingenberg, C. P. (2021). How exactly did the nose get that long? A critical rethinking of the Pinocchio effect and how shape changes relate to landmarks. Evolutionary Biology, 48(1), 115–127. 10.1007/s11692-020-09520-y

[ece311588-bib-0042] Klingenberg, C. P. , & Marugán‐Lobón, J. (2013). Evolutionary covariation in geometric morphometric data: Analyzing integration, modularity, and allometry in a phylogenetic context. Systematic Biology, 62(4), 591–610. 10.1093/sysbio/syt025 23589497

[ece311588-bib-0043] Kraatz, B. , & Sherratt, E. (2016). Evolutionary morphology of the rabbit skull. PeerJ, 4, e2453. 10.7717/peerj.2453 27688967 PMC5036099

[ece311588-bib-0044] Legendre, P. , & Legendre, L. (2012). Numerical ecology (3rd English Edition ed.). Elsevier.

[ece311588-bib-0045] Lessa, E. P. , & Patton, J. L. (1989). Structural constraints, recurrent shapes, and allometry in pocket gophers (genus Thomomys). Biological Journal of the Linnean Society, 36(4), 349–363. 10.1111/j.1095-8312.1989.tb00500.x

[ece311588-bib-0046] Marcy, A. E. , Fruciano, C. , Phillips, M. J. , Mardon, K. , & Weisbecker, V. (2018). Low resolution scans can provide a sufficiently accurate, cost‐ and time‐effective alternative to high resolution scans for 3D shape analyses. PeerJ, 6, e5032. 10.7717/peerj.5032 29942695 PMC6016532

[ece311588-bib-0047] Marcy, A. E. , Guillerme, T. , Sherratt, E. , Rowe, K. C. , Phillips, M. J. , & Weisbecker, V. (2020). Australian rodents reveal conserved cranial evolutionary allometry across 10 million years of murid evolution. The American Naturalist, 196(6), 755–768. 10.1086/711398 33211559

[ece311588-bib-0048] Marcy, A. E. , Hadly, E. A. , Sherratt, E. , Garland, K. , & Weisbecker, V. (2016). Getting a head in hard soils: Convergent skull evolution and divergent allometric patterns explain shape variation in a highly diverse genus of pocket gophers (*Thomomys*). BMC Evolutionary Biology, 16(1), 207. 10.1186/s12862-016-0782-1 27724858 PMC5057207

[ece311588-bib-0049] Marroig, G. , & Cheverud, J. M. (2005). Size as a line of least evolutionary resistance: Diet and adaptive morphological radiation in new world monkeys. Evolution, 59(5), 1128–1142. 10.1111/j.0014-3820.2005.tb01049.x 16136810

[ece311588-bib-0050] McIntosh, A. F. , & Cox, P. G. (2016). The impact of digging on craniodental morphology and integration. Journal of Evolutionary Biology, 29(12), 2383–2394. 10.1111/jeb.12962 27521516

[ece311588-bib-0051] Mitchell, D. R. (2019). The anatomy of a crushing bite: The specialised cranial mechanics of a giant extinct kangaroo. PLoS One, 14(9), e0221287. 10.1371/journal.pone.0221287 31509570 PMC6738596

[ece311588-bib-0052] Mitchell, D. R. , Potter, S. , Eldridge, M. D. , Martin, M. , & Weisbecker, V. (2024). Functionally mediated cranial allometry evidenced in a genus of rock‐wallabies. Biology Letters, 20(3), 20240045. 38531413 10.1098/rsbl.2024.0045 PMC10965333

[ece311588-bib-0053] Mitchell, D. R. , Sherratt, E. , Ledogar, J. A. , & Wroe, S. (2018). The biomechanics of foraging determines face length among kangaroos and their relatives. Proceedings of the Royal Society B: Biological Sciences, 285(1881), 20180845. 10.1098/rspb.2018.0845 PMC6030537 29925620

[ece311588-bib-0054] Mitchell, D. R. , Sherratt, E. , & Weisbecker, V. (2024). Facing the facts: Adaptive trade‐offs along body size ranges determine mammalian craniofacial scaling. Biological Reviews, 99(2), 496–524. 10.1111/brv.13032 38029779

[ece311588-bib-0055] Murray, B. R. , Dickman, C. R. , Watts, C. H. S. , & Morton, S. R. (1999). The dietary ecology of Australian rodents. Wildlife Research, 26(6), 857–858. 10.1071/WR97046_CO

[ece311588-bib-0056] Oksanen, J. , Simpson, G. , Blanchet, F. , Kindt, R. , Legendre, P. , Minchin, P. , O'Hara, R. , Solymos, P. , Stevens, M. , Szoecs, E. , Wagner, H. , Barbour, M. , Bedward, M. , Bolker, B. , Borcard, D. , Carvalho, G. , Chirico, M. , De Caceres, M. , Durand, S. , … Weedon, J. (2022). vegan: Community Ecology Package. *R package version 2.6‐4* https://CRAN.R‐project.org/package=vegan

[ece311588-bib-0057] R Core Team . (2024). R: A language and environment for statistical Computing_. R Foundation for Statistical Computing. https://www.R‐project.org/

[ece311588-bib-0058] Radinsky, L. (1985). Approaches in evolutionary morphology: A search for patterns. Annual Review of Ecology and Systematics, 16(1), 1–14.

[ece311588-bib-0059] Samuels, J. X. (2009). Cranial morphology and dietary habits of rodents. Zoological Journal of the Linnean Society, 156(4), 864–888. 10.1111/j.1096-3642.2009.00502.x

[ece311588-bib-0060] Satoh, K. , & Iwaku, F. (2006). Jaw muscle functional anatomy in northern grasshopper mouse, Onychomys leucogaster, a carnivorous murid. Journal of Morphology, 267(8), 987–999. 10.1002/jmor.10443 16710844

[ece311588-bib-0061] Schlis‐Elias, M. C. , & Malaney, J. L. (2022). Island biogeography predicts skull gigantism and shape variation in meadow voles Microtus pennsylvanicus through ecological release and allometry. Oikos, 2022(4), e08777. 10.1111/oik.08777

[ece311588-bib-0062] Segura, V. , Flores, D. , & Deferrari, G. (2023). Comparison of skull growth in two ecosystem modifiers: Beavers *Castor canadensis* (Rodentia: Castoridae) and muskrats *Ondatra zibethicus* (Rodentia: Cricetidae). Zoologischer Anzeiger, 304, 61–72.

[ece311588-bib-0063] Singleton, M. (2005). Functional shape variation in the Cercopithecine masticatory complex. In D. E. Slice (Ed.), Modern Morphometrics in physical anthropology (pp. 319–348). Springer US.

[ece311588-bib-0064] Smaers, J. B. , Rothman, R. S. , Hudson, D. R. , Balanoff, A. M. , Beatty, B. , Dechmann, D. K. N. , de Vries, D. , Dunn, J. C. , Fleagle, J. G. , Gilbert, C. C. , Goswami, A. , Iwaniuk, A. N. , Jungers, W. L. , Kerney, M. , Ksepka, D. T. , Manger, P. R. , Mongle, C. S. , Rohlf, F. J. , Smith, N. A. , … Safi, K. (2021). The evolution of mammalian brain size. Science Advances, 7(18), eabe2101. 10.1126/sciadv.abe2101 33910907 PMC8081360

[ece311588-bib-0077] Smissen, P. J. , & Rowe K. C. (2018). Repeated biome transitions in the evolution of Australian rodents. Molecular Phylogenetics and Evolution, 128, 182–191. 30075296 10.1016/j.ympev.2018.07.015

[ece311588-bib-0065] Strait, D. S. , Weber, G. W. , Neubauer, S. , Chalk, J. , Richmond, B. G. , Lucas, P. W. , Spencer, M. A. , Schrein, C. , Dechow, P. C. , Ross, C. F. , Grosse, I. R. , Wright, B. W. , Constantino, P. , Wood, B. A. , Lawn, B. , Hylander, W. L. , Wang, Q. , Byron, C. , Slice, D. E. , & Smith, A. L. (2009). The feeding biomechanics and dietary ecology of *Australopithecus africanus* . Proceedings of the National Academy of Sciences, 106(7), 2124–2129. 10.1073/pnas.0808730106 PMC2650119 19188607

[ece311588-bib-0066] Tamagnini, D. , Meloro, C. , & Cardini, A. (2017). Anyone with a long‐face? Craniofacial evolutionary allometry (CREA) in a family of short‐faced mammals, the Felidae. Evolutionary Biology, 44(4), 476–495.

[ece311588-bib-0067] Tamagnini, D. , Michaud, M. , Meloro, C. , Raia, P. , Soibelzon, L. , Tambusso, P. S. , Varela, L. , & Maiorano, L. (2023). Conical and sabertoothed cats as an exception to craniofacial evolutionary allometry. Scientific Reports, 13(1), 13571. 37604901 10.1038/s41598-023-40677-6 PMC10442348

[ece311588-bib-0068] Van Valkenburgh, B. (1989). Carnivore dental adaptations and diet: A study of trophic diversity within guilds. In J. L. Gittleman (Ed.), Carnivore behavior, ecology, and evolution (pp. 410–436). Springer US.

[ece311588-bib-0069] Voje, K. L. , Hansen, T. F. , Egset, C. K. , Bolstad, G. H. , & Pélabon, C. (2014). Allometric constraints and the evolution of allometry. Evolution, 68(3), 866–885. 10.1111/evo.12312 24219593

[ece311588-bib-0070] Watts, C. , & Kemper, C. (1989). Muridae. Fauna of Australia, 1, 939–956.

[ece311588-bib-0071] Weisbecker, V. , Guillerme, T. , Speck, C. , Sherratt, E. , Abraha, H. M. , Sharp, A. C. , Terhune, C. E. , Collins, S. , Johnston, S. , & Panagiotopoulou, O. (2019). Individual variation of the masticatory system dominates 3D skull shape in the herbivory‐adapted marsupial wombats. Frontiers in Zoology, 16(1), 41. 10.1186/s12983-019-0338-5 31695725 PMC6824091

[ece311588-bib-0072] Weisbecker, V. , Rowe, T. , Wroe, S. , Macrini, T. E. , Garland, K. L. S. , Travouillon, K. J. , Black, K. , Archer, M. , Hand, S. J. , Berlin, J. C. , Beck, R. M. D. , Ladevèze, S. , Sharp, A. C. , Mardon, K. , & Sherratt, E. (2021). Global elongation and high shape flexibility as an evolutionary hypothesis of accommodating mammalian brains into skulls. Evolution, 75(3), 625–640. 10.1111/evo.14163 33483947

[ece311588-bib-0073] Williams, S. H. , Peiffer, E. , & Ford, S. (2009). Gape and bite force in the rodents *Onychomys leucogaster* and *Peromyscus maniculatus*: Does jaw‐muscle anatomy predict performance? Journal of Morphology, 270(11), 1338–1347. 10.1002/jmor.10761 19480012

[ece311588-bib-0074] Wilson, L. A. B. (2013). Allometric disparity in rodent evolution. Ecology and Evolution, 3(4), 971–984. 10.1002/ece3.521 23610638 PMC3631408

[ece311588-bib-0075] Winkler, D. E. , Schulz‐Kornas, E. , Kaiser, T. M. , De Cuyper, A. , Clauss, M. , & Tütken, T. (2019). Forage silica and water content control dental surface texture in Guinea pigs and provide implications for dietary reconstruction. Proceedings of the National Academy of Sciences, 116(4), 1325–1330. 10.1073/pnas.1814081116 PMC6347716 30606800

[ece311588-bib-0076] Zelditch, M. L. , & Swiderski, D. L. (2023). The predictable complexity of evolutionary allometry. Evolutionary Biology, 50(1), 56–77.

