# Supplementary material for: Beyond CREA: Evolutionary patterns of non‐allometric shape variation and divergence in a highly allometric clade of murine rodents
Source: Ecol Evol. 2024 Jun 28;14(7):e11588. doi: 10.1002/ece3.11588 (PMC11213820; doi:10.1002/ece3.11588)
Supplement: Supplementary file 1 — Data S1. [file ECE3-14-e11588-s001.zip › Supp1_LM_heatmap_comparisons.pdf]

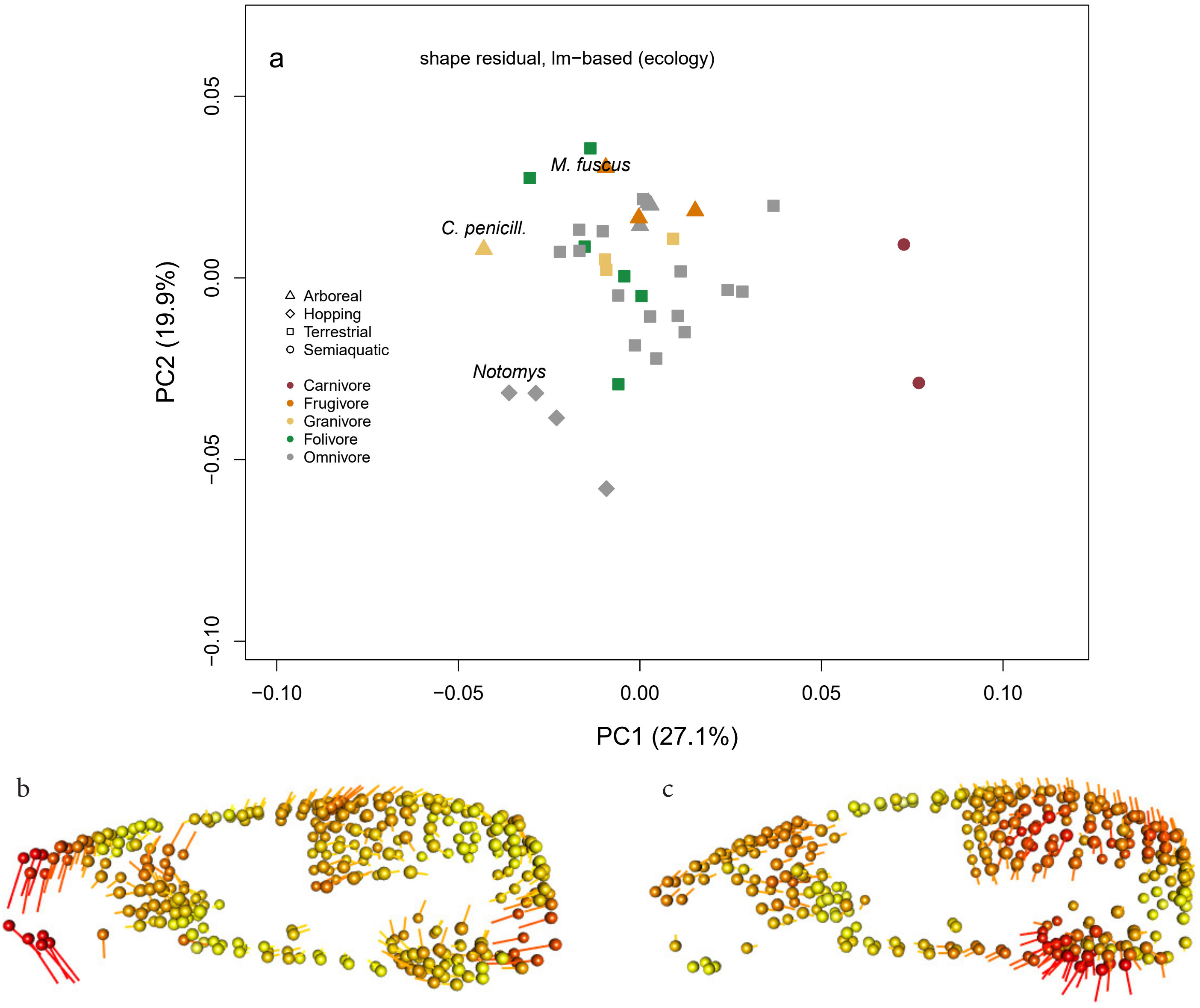

Supplementary Figure 1: Shape variation after size removal based on a non-phylogenetically corrected linear regression of shape against log(centroid size) a, Plot of PC1 and PC2 for the residuals; b, shape variation from high(balls) to low (end of hairs) PC1 scores; c, shape variation from high (balls) to low (ends of hairs) PC2 scores. Spheres show the mean position of landmarks for the column's dataset, vectors show landmark displacement. Colors and lengths are calculated from relative proportions of the minimum/maximum vector lengths for each comparison,and are not equivalent across individual images
