# Supplementary material for: Beyond CREA: Evolutionary patterns of non‐allometric shape variation and divergence in a highly allometric clade of murine rodents
Source: Ecol Evol. 2024 Jun 28;14(7):e11588. doi: 10.1002/ece3.11588 (PMC11213820; doi:10.1002/ece3.11588)
Supplement: Supplementary file 1 — Data S1. [file ECE3-14-e11588-s001.zip › Supplementary Table 1_integration_pvalues.docx]

**Supplementary Table 1: P**hylogenetically informed integration, expressed as r-PLS values, between the full shape (top) and residual shape (bottom) of modules. r-PLS values are in the bottom triangle, probability values are in the top triangles. Numbers in the diagonal are landmark numbers for each partition.

|  | Basicranium | Molar | Orbital | Rostrum | Vault |
| --- | --- | --- | --- | --- | --- |
| Basicranium | 64 | 0.001 | 0.001 | 0.001 | 0.001 |
| Molar | 0.69 | 19 | 0.002 | 0.001 | 0.001 |
| Orbital | 0.77 | 0.7 | 32 | 0.001 | 0.001 |
| Rostrum | 0.89 | 0.77 | 0.86 | 86 | 0.001 |
| Vault | 0.85 | 0.77 | 0.88 | 0.94 | 124 |

|  | Basicranium | Molar | Orbital | Rostrum | Vault |
| --- | --- | --- | --- | --- | --- |
| Basicranium | 64 | 0.011 | 0.001 | 0.003 | 0.001 |
| Molar | 0.59 | 19 | 0.074 | 0.001 | 0.003 |
| Orbital | 0.8 | 0.52 | 32 | 0.001 | 0.001 |
| Rostrum | 0.71 | 0.82 | 0.76 | 86 | 0.001 |
| Vault | 0.88 | 0.69 | 0.85 | 0.79 | 124 |
